# The Expert Patient and Chronic Respiratory Diseases

**DOI:** 10.1155/2016/9454506

**Published:** 2016-04-14

**Authors:** Louis-Philippe Boulet

**Affiliations:** Institut Universitaire de Cardiologie et de Pneumologie de Québec, Université Laval, Québec, QC, Canada G1V 3G7

## Abstract

The concept of “expert patient” has been developed in the last two decades to define a patient who has a significant knowledge of his/her disease and treatment in addition to self-management skills. However, this concept has evolved over the last years, and these patients are now considered, not only to be more efficient in the management of their own condition and communicating effectively with health professionals, but to also act as educators for other patients and as resources for the last, provide feedback on care delivery, and be involved in the production and implementation of practice guidelines, as well as in the development and conduct of research initiatives. There are some barriers, however, to the integration of this new contributor to the health care team, and specific requirements need to be considered for an individual to be considered as an expert. This new player has, however, a potentially important role to improve current care, particularly in respiratory health.

## 1. The Burden of Chronic Diseases in Canada

Diseases such as asthma, chronic obstructive pulmonary disease (COPD), diabetes, and many other chronic conditions represent an increasing burden for respiratory health systems. For example, loss of productivity in Canada in 2010 due to the morbidity associated with respiratory diseases was estimated to be 117 million dollars [1]. As most of these conditions ideally require an involvement of the patient and his peers in their management, it is considered essential to provide the last with the educational interventions required to adequately evaluate their condition and apply the treatments proposed. Furthermore, the severity or control of these conditions can change over time. For example, exacerbations of airways diseases may follow respiratory infections or various environmental exposures. The patient, therefore, needs to know how to adapt his treatment and apply preventative measures.

## 2. Patient Education and Self-Management: A Necessity

What is called “therapeutic education,” aiming to improve knowledge and self-management or comanagement of a chronic disease, is a universal recommendation of current guidelines. This is particularly true for asthma and COPD, although, for the last, how these interventions should be applied and what should be the content of these interventions is still a matter of debate. According to Wilson et al., a better understanding of patients' educational needs should allow health professionals to optimize the effects of readaptation or educational programs for COPD, in integrating essential notions in a format that is acceptable for the patients [2]. According to the last Canadian guidelines on COPD, educational interventions should be well articulated and provide a mean to reduce the effects of the disease in a majority of patients [3].

For asthma, Gibson et al. showed that the benefits provided through patient education and self-management included an improvement in quality of life and in asthma control, in addition to a reduction in hospital admissions, emergency visits, rescue bronchodilator use, need for oral corticosteroids, and number of urgent medical visits [4]. Unfortunately, the number of patients referred to education centers or to local educators is still too low in Canada as in other countries, and the time that physicians, particularly in primary care, have available for this type of intervention is usually quite limited [5]. There is also a lack of accessibility to trained educators and a lack of knowledge about how and where to refer the patient.

Efforts should therefore be done to improve access to high-quality patient education not only in specialized centers but also in primary care settings. In this regard, we recently performed a study on the effects of providing educational interventions to patients followed up in family medicine clinics (groupe de médecine familiale—GMF) in their own environment. It showed that, in addition to improving knowledge of asthma patients, it could reduce unscheduled medical visits and lead to a better use of medications [6].

Furthermore, although educational interventions are ideally done by trained educators, following their initiation by the physician, both the physician and the educator can also take part in shared-decision making (SDM), following the transfer of basic knowledge to the patient and acquisition of self-management skills. Indeed, recent studies on SDM have shown the benefits of involving the patient in treatment decisions, improving patient confidence, and reducing decisional conflicts [7]. For asthma, as an example, SDM improved adherence to treatment and clinical outcomes [8]. This approach is a change from the paternalistic medical approach towards a bidirectional exchange, allowing obtaining an informed decision from the patient, hopefully improving the possibility to increase adherence to recommendations, and significantly improving clinical outcomes.

In recent years, patients' role in disease management has not been restricted to their own care, but they have also been considered as potential deliverers of care or, at least, contributors to improve care delivery and research initiatives. From this, the concept of “expert patient” has emerged [9–11].

## 3. Benefits Associated with Expert Patients

With an increasing access to information and educational programs, expert patients, even if they have been around for a long time, are increasing in numbers. These patients have a large potential to improve care, particularly for chronic diseases and rare conditions. Expert patients may help health professionals to make appropriate decisions and to improve quality of care. Examples of the possible roles of the expert patients are found in Table 1 and will be discussed in the following lines.

### 3.1. Improving Self-Management of Chronic Diseases

When we look at the definition of an expert patient, we generally find some characteristics that include the acquisition of significant knowledge and skills in a particular domain. Understanding the essential characteristics of a disease and its management and developing communication skills are part of the requirements to become an expert. Recent publications suggest reserving this term for patients who at least (1) want to understand the nature of their health problem and its treatment and wish to use and update their knowledge appropriately; (2) achieve control of their condition; and (3) want to contribute to the management of their disease in partnership with health professionals with whom they should communicate effectively [9–12] (Table 1).

We can consider, however, that there are many levels of patient's expertise and that there are various types of expert patients. For example, a health professional may suffer from a disease not in his or her field of practice and want to acquire additional knowledge to intervene in this domain of health care.

A patient's family member, sometimes already involved in helping this person, may want to improve his or her skills and knowledge to better support the former. This may help the patient to better use treatments, improve adherence, and facilitate communications with health care givers.

A patient may also want to acquire a good knowledge about his disease without being necessarily an expert. If the knowledge is significant and is applied into care regularly, a certain degree of expertise can nevertheless be achieved. Physicians occasionally acknowledge that some of their patients know their health problems as well as or even better than themselves. With appropriate support, it is recognized that most people suffering from a chronic disease can take responsibility of their disease management, guided by health professionals. This usually results in an improvement of their condition and quality of life.

### 3.2. The Evolving Concept of Expert Patient

We are just at the beginning of better defining the concept of expert patient and this should still change over time. Indeed, Cordier recently suggested the necessity to revise this notion and reminds us that this updated concept has been initially promoted in 1999 in a report presented to the British Parliament wishing to find a solution to the increasing problem of chronic diseases [12, 13]. This was inspired by the work of Lorig, at the Chronic Disease Self-Management Programs (CDSMP) in Stanford, California [14, 15]. A subsequent report of the UK National Health Service suggested the development of an expert patient program based on the development of confidence and motivation to use their knowledge and skills to achieve a better control of their disease [14]. This program wished to improve awareness of health problems, to promote patient's expertise in care, and to better inform health professionals about what should be included in comanagement programs led by care users.

### 3.3. The Patient Acting as an Educator and Collaborator

Although the expert patient's main role would be to better self-manage their disease, it has been suggested that they could also lead educational programs for fellow patients. However, Newman et al. reported in 2004, after reviewing 62 studies on conditions such as type 2 diabetes, arthritis, and asthma, that only three were lay-led self-management programs, the others being led by health care professionals [16].

Not only can the expert patient contribute to educating other patients, but he/she can also help update knowledge of health professionals and participate in the development and evaluation of educational programs or guidelines, as well as participate in communities of practice and web-based patient-targeted communications. Identification of patient needs and communication of their values, perceptions, and preferences can help improve management strategies, particularly in specific populations such as immigrants, elderly, or patients from various cultural backgrounds.

Expert patients have recently been integrated into programs to play the role of educators for other patients. In a review of 17 studies involving 7442 participants, Foster et al. showed that this type of intervention could improve, at least on a short-term basis, self-efficacy, autoevaluated health status of the patients, and management of symptoms, in addition to increasing the frequency of aerobic exercises [17].

Looking at delivery of self-management education in primary care, Partridge et al. also suggested, from a study of 567 patients randomized to care by a nurse or a lay educator, that it is possible to recruit and train lay educators to deliver a discrete area of respiratory care, with comparable outcomes to those seen by nurses [18].

In Canada, the University of British Columbia Interprofessional Health Mentors Program has recently described its 3-year pilot program as an elective patient-as-teacher initiative in which groups of four students from different disciplines learn together with a mentor suffering from a chronic condition—an “expert by experience”—over three semesters. Students and mentors rated the program highly, and a wide range of important learning outcomes have been documented [19]. This program remains, however, to be further evaluated in regard to other outcomes.

### 3.4. Involvement of the Expert Patient in Research and Development

Research-funding bodies increasingly require the participation of patients to research initiatives and promote “patient-oriented” research (POR). POR usually refers to a continuum of research ranging from the initial human studies of a new drug or device to research that evaluates the implementation of interventions in the health care system. It includes the evaluation of new and current diagnostic approaches treatments, devices, or practices, as well as the synthesis, dissemination, and integration of this new knowledge into care [20]. The promotion of “integrated knowledge translation” in which patient-relevant studies must include patients at each stage of their design and various aspects of the project can benefit from their input and hopefully make the results more directly implementable.

#### 3.4.1. Barriers and Difficulties with the Development of Expert Patients

There are many barriers to the development of expert patients [21, 22]. First, health care professionals could fear that this type of initiative will increase the workload and require more time during interventions. Physicians can consider that patients risk knowing more than them and challenge their expertise and decisions. The physicians may also not be interested to take part in SDM interventions.

The physician can fear that the patient could become a pseudoexpert and that the information acquired is not necessarily exact, evidence-based, or relevant. As an example, there is so much wrong or inappropriate information on the internet and in the media, sometimes conveying misconceptions and notions not based on evidence, applicable to very selective types of patients, commercially biased, or simply wrong. As this is a potential problem, measures such as those suggested in Table 2 should ideally be implemented, so that health professionals can gain confidence in the expert patient's abilities. In regard to factors associated with the patient, he/she may have insufficient confidence in regard to his/her capacity to play this role. Many patients do not want to become an expert due to other priorities, lack of time, insufficient literacy, or simply passivity.

#### 3.4.2. Evaluation of Expert Patients Programs

Newbould et al. [23] previously reported and reviewed expert patient programs in the United Kingdom and the literature on lay-led self-management in chronic illness and warned that despite the potential benefits, at least short-term benefits, of enhanced self-management, possible problems can be associated with the presentation and implementation of such initiatives. These can include overstating the evidence for effectiveness of the programs or provision of too rigid prescriptions about what patients should think and do [23]. For some of the studies reported, the “intervention” group either was compared to usual care, mailing of information or had no control group (pre- and postinterventions). The benefit of expert patient programs should therefore be further assessed and it will be helpful to contextualize those by comparing the last to the effects of professional-led interventions. Indeed, expert patients programs are increasingly evaluated in regard to their effect on the patient, showing significant improvements in patient's self-efficacy, but much remains to be known on their effects on global health care and how to optimize the last.

Many educational programs contribute to developing such experts but are not necessarily called “expert programs.” The initial so-called “expert patient” programs show a certain heterogeneity and their goals were mostly at the level of improving the patient's own condition. Among those various programs, for example, we find the Glaucoma Expert Patient Program, a glaucoma-specific educational self-management program aimed at improving glaucoma patients' knowledge, self-management skills, expectations, and adherence to treatment [24], and the expert patient self-management programs on bronchiectasis and Stanford University programs on chronic diseases [25, 26]. A study by Lorig et al. looking at the effectiveness of an online self-management program (EPP Online) for England residents with long-term conditions showed that it was associated with a significant improvement at 6 months for all variables except self-rated health, disability, hospitalizations, and nights in hospital [27]. At 12 months only, decrease in disability, nights in hospital, and hospitalizations were not significant. Both self-efficacy and satisfaction with the health care system improved significantly.

In regard to the evaluation of peer-led-programs, the effectiveness of an online self-management program for patients with long-term conditions showed improvements to some extent in many aspects of the conditions and reduced health care use while self-efficacy and satisfaction with the health care system improved significantly [28].

Globally, few will doubt about potential benefits of an informed patient, ideally with a certain degree of expertise, in managing his/her disease but there is a need to go one step further and assess other benefits of providing a higher level of expertise for patients. This should be counterbalanced with the need for resources allocated to such programs. Furthermore, the influence of patients on educational programs or research projects remains to be evaluated, including their cost-benefits.

#### 3.4.3. Some Elements to Consider about Expert Patients Programs

Patient education, additional training, and updates should be based on evidence and the most recent guidelines recommendations to ensure quality of information (Table 2). This also requires a close collaboration and communication with health care teams. New skills may need to be acquired. Ideally, the patient should have an experienced mentor, such as a medical specialist in this field, and, if available, follow an accreditation program, such as those offered to educators. Participation in a committee of practice may help.

Current practice guidelines produced by recognized organizations such as thoracic societies could be ideally translated into a format targeting the patient while including the last in their production, but the information should be adapted to the patients who have no background in health care. Recommendations should be easy to understand. There have been, however, difficulties to accomplish this task and with the growing need for effective knowledge translation, particularly with electronic communications and social networks, patient engagement, and patient-oriented research, we need expert patients to fulfill these roles.

#### 3.4.4. Financing Experts Programs

Some patients will work without remuneration and may be willing to pay for their training, but, in many instances, they will need support to cover those activities. This could be integrated into current educational programs, institutions, and activities and/or supported by patients' or other health professional organizations. Cost-benefit of these interventions remains to be determined.

#### 3.4.5. Development of Tools for Expert Patients

Many tools have been developed to help patients become experts in their disease. Medical organizations, as well as local institutions, have produced such aids and programs. As an example, the Laval University Chair of Knowledge, Education, Prevention, and Knowledge Translation in Respiratory and Cardiovascular Health are developing a series of publications for patients based on the concept of “expert patient” on various respiratory diseases in both paper and electronic format at low cost, in addition to audiovisual materials (http://www.coeurpoumons.ca/). These tools may help patients to become experts in their own care but of course they will require contact with educators and physicians to ensure that their training is adequate if they want to become involved at another level.

## 4. Conclusions

The expert patient has a role to play in the management of chronic diseases, not only for a better management of his/her condition, but also for the development of educational programs and guidelines in addition to improving care delivery and contributing to research initiatives. More should be known, however, on how this new player could optimize its contribution to current health initiatives and how to ensure sufficient quality of their interventions and regular update.

## Figures and Tables

**Table 1:** Domains and some possible roles of expert patients.

Clinical	Help disease management (for themselves and/or other patients, particularly for chronic or rare conditions) Identification of patient needs and communication of their values, perceptions, and preferences Identify and report on possible side effects of treatments Support interventions from physicians and other health care professionals, particularly in primary care—help translation of guidelines into care

Educational	Education of other patients Contribute to updating knowledge of health professionals Help develop decision aids Contribute to the development and evaluation of educational programs Participate in patients' versions of guidelines or directly contribute to current medical guidelines Get involved in an internet chat or blog or in a community of practice

Research	Advise on study designs Contribute to evaluation of new treatments Help choose research questions and define patient-relevant outcomes Help to design and implement end-of-study knowledge translation plans

Others	Lobby to health care authorities Represent patients in various committees Participate in activities of patients' associations Contribute to the development of support groups

**Table 2:** Requirements to become (and remain) an expert patient.

Motivation	Willing to get involved in the process of becoming an expert patient Motivated to participate in care delivery/improvement

Training and update	Acquire basic education in the domain selected Ideally take part in a certification program whenever available Ensure regular update of knowledge and skills Benefit from an active mentorship and effectively communicate with experts Proceed regularly to a formal evaluation of competences Participate in meetings and conferences in the field

Avoid biases	Avoid any commercial influences or personal biases in interventions Always check sources of information Avoid acting in domains out of field of expertise
